# Do Phagostimulants, Alone or Combined with Ammonium Acetate, Di-Ammonium Phosphate, and Acetic Acid, Effectively Attract Both Sexes of Peach Fruit Flies, *Bactrocera zonata* (Diptera: Tephritidae)?: Insights from Laboratory and Field Bioassays

**DOI:** 10.3390/insects15070470

**Published:** 2024-06-24

**Authors:** Muhammad Junaid Nisar, Muhammad Dildar Gogi, Asim Abbasi, Bilal Atta, Qudsia Yousafi, Inzamam Ul Haq, Mishal Subhan, Hayssam M. Ali, Waleed A. A. Alsakkaf, Mohamed S. Basiouny

**Affiliations:** 1 Integrated Pest Management Laboratory, Department of Entomology, University of Agriculture, Faisalabad 38040, Pakistan; 2 Department of Entomology, University of Agriculture, Faisalabad 38040, Pakistan; asimuaf95@gmail.com; 3 Rice Research Institute, Kala Shah Kaku 39020, Pakistan; 4 Department of Biosciences, COMSATS University Islamabad, Sahiwal Campus, Sahiwal 57000, Pakistan; 5 State Key Laboratory of Ecological Pest Control for Fujian and Taiwan Crops, Key Laboratory of Biopesticide and Chemical Biology, MOE, College of Plant Protection, Fujian Agriculture and Forestry University, Fuzhou 350002, China; 6 Department of Microbiology and Molecular Genetics, The Women University Multan, Multan 66000, Pakistan; 7 Department of Botany and Microbiology, College of Science, King Saud University, Riyadh 11451, Saudi Arabiawalsakkaf.c@ksu.edu.sa (W.A.A.A.); 8 School of Biotechnology, Badr University in Cairo (BUC), Badr City, Cairo 11829, Egypt

**Keywords:** *Bactrocera zonata*, attractancy, phagostimulants, phagostimulant lure sources, ammonium acetate, dap, acetic acid

## Abstract

**Simple Summary:**

This study evaluated the effectiveness of various attractants, including phagostimulants alone or combined with other chemicals, in attracting both male and female *Bactrocera zonata* in laboratory and field experiments. Different mixtures of natural fruit extracts and synthetic attractants were tested to find the most effective combination for trapping *B. zonata*. The results indicated that a specific combination (phagostimulant-AdMixture-4) was particularly effective, showing strong attraction capabilities in both laboratory and field settings. These findings were significant and suggested a potential new method for managing fruit fly populations, which are a major cause of fruit damage and economic loss in different fruit orchards. The implementation of such attractant systems could reduce the need for chemical pesticides, offering an eco-friendly and sustainable solution to pest control in different fruit production systems. This advancement holds promise not only for increasing the yield and quality of fruit but also for supporting safer and more sustainable agricultural practices.

**Abstract:**

Laboratory and field assays of three sets of experiments were conducted to evaluate the impact of different phagostimulants alone and in combination with other phagostimulant lure sources, such as ammonium acetate, DAP, and acetic acid, on the attractancy of both sexes of *B. zonata*. In the first experiment, the laboratory olfactometer study revealed that out of eleven phagostimulants, banana, mulberry, mango, guava, molasses, and protein hydrolysate exhibited moderate attractancy (15.2–60.2%) to *B. zonata*. Unexpectedly, banana and protein hydrolysate were demonstrated to be highly attractive phagostimulants for starved female *B. zonata* (53.6% and 60.2%, respectively). In the field study, none of the tested phagostimulants exhibited high attractancy; however, banana, mulberry, protein hydrolysate, guava, mango, and molasses demonstrated moderate attractancy (5.6–35.6%) to *B. zonata*. In the second experiment, out of five phagostimulant-mixtures, phagostimulant-mixture-4 proved highly attractive (40.5–68.6% and 45.5–51.2%), followed by phagostimulant-mixture-3, which proved to be moderately attractive (17.0–22.5% and 28.4–36.1%) to *B. zonata* in olfactometer and field studies, respectively. In the third experiment, out of five phagostimulant-AdMixtures, phagostimulant-AdMixture-4 demonstrated strong attractiveness in the olfactometer (41.6–68.7%) and field studies (52.7–58.7%) for *B. zonata*, while the rest of the AdMixtures demonstrated moderate to no attractiveness for *B. zonata*. So, phagostimulant-AdMixture-4 with GF-120 could be used in the development of a phagostimulant bait station which attracts the maximum *B. zonata* population and ultimately provides pest-free fruits to the farmers

## 1. Introduction

The low yield of fruits is attributed to many factors, including insect pests [1,2,3,4,5,6], diseases [7,8,9,10,11,12], alternate bearing [13], harvesting and storage issues [14,15], improper packing [16], handling and transportation [17,18] and postharvest losses [19]. All these problems have been reported as the most important limiting factors in the production and export of fruits, especially mango, and they cause quarantine issues [20,21,22].

*Bactrocera zonata* (Diptera: Tephritidae) causes both qualitative and quantitative losses of fruits and vegetables [22]. This particular fruit fly is one of the severe quarantine pest species, and it puts pressure on the scenario of international markets dealing in horticultural produce [23,24]. It also causes both direct and indirect losses of horticultural crops. Direct damage is caused when female *B. zonata* ovi-puncture the fruits for oviposition; then, larvae emerge from eggs and feed cryptically on the fruit pulp [24,25].

Food baits are considered to be a vital strategic component of the insect trapping system and are mostly used for monitoring the outbreak and infestation of tephritid fruit flies [26,27,28]. Conventionally, the most frequently used food bait trap was the torula yeast borax trap used against tephritid fruit flies. Later on, a hydrolyzed animal protein-based food bait trap (Cera Trap) proved to be a better alternate to the torula yeast trap [27]. The attract-and-kill efficacy of malathion bait spray was evaluated in the field against *C. capitata* and had very encouraging attract-and-kill results, and lower fruit damage was recorded in a citrus orchard when compared to a magnet bait station [29]. The relative attractancy of food baits to fruit flies varies interspecifically [27]. Medfly captures have been recorded as being significantly higher in the Cera Trap compared to the torula yeast–borax-baited traps [26]. Similarly, Shelly and Kurashima [27] reported that the torula yeast trap proved more effective against *Zeugodacus cucurbitae* (Coquillett) compared to *B. dorsalis*, while both the torula yeast trap and the Cera Trap demonstrated significantly higher captures of both fruit fly species and proved equally effective against *B. dorsalis* and *Z. cucurbitae* [27].

The effectiveness of food bait is highly dependent on the concentration of food attractants used in the food bait [30] and the pH of the bait [31]. Food baits (Bio-Nal and Buminal) of different types are used to attract and kill female and male pestiferous fruit flies. *C. capitata* were more attracted to Buminal baits; however, Cera Traps proved more attractive to *B. zonata* compared to *C. capitata*. The fly density captured in traps was highly affected by the pH [31]. Six protein hydrolysate-based food baits at different concentrations proved highly effective against both male and female melon fly, *B. cucurbitae*, while the effectiveness was directly proportional to the concentration [30]. Protein- and Spinosad-based attractants, along with two different parasitoids, demonstrated significant results and proved to be very effective against fruit flies. Similarly, the application of Spinosad and bait sprays of GF-120 demonstrated a 47 to 63% reduction in the population of *Anastrepha ludens* in comparison with control orchards [32].

A number of different food baits were evaluated for the attraction to fruit flies in mango and guava orchards. Proteinex (a formulated protein supplement that includes a mixture of proteins and other nutrients, like amino acids, vitamins, and minerals) and an ammonium acetate (5%)-containing food trap attracted 5.17, 9.42, and 2.25 adults of *B. correcta, B. dorsalis*, and *B. cucurbitae* trap^−1^ week^−1^, respectively. The integration of ammonium acetate along with acetic acid (5%), casein, proteinex, and mango pulp proved to be the most effective treatment for managing fruit flies [33]. The evaluation results of some attractants revealed that populations of female *B. zonata* and *C. capitata* were not affected by the application of protein hydrolysate and GF-120 (an environmentally friendly fruit fly bait containing Spinosad, which is derived from a naturally occurring soil bacterium, *Saccharopolyspora spinosa*) baits; however, the combination of baits with some attractive additives enhanced their attraction to fruit flies [34]. A waste brewer’s yeast trap with two components, i.e., trimethylamine and ammonium acetate, enhanced the attraction of the trap and almost 75% of the fruit flies were captured in such traps, which was found to be significantly higher than the brewer’s yeast trap [34]. Similarly, protein hydrolyzed bait (GF-120) at its higher concentration proved to be a very effective tool for fruit fly management [35].

GF-120 at its high, moderate, and low dose was evaluated for its efficacy against fruit flies. The results revealed that fewer fruit flies were trapped at low and moderate doses, while significantly more fruit flies were captured in GF-120 at a high dose [36]. According to Flores et al. [37], GF-120, when assessed at different concentrations (24, 48, 72, and 96 ppm) against *Anastrepha obliqua*, *A. serpentine*, and *A. ludens*, demonstrated significantly higher mortality of three fruit fly species at a 24 ppm concentration. The application of GF-120 at a 72 ppm concentration proved effective against these fruit fly species in the field for minimum period of 10 days [37]. Royer et al. [38] documented that the Cera Trap captured less females of *B. cucumis* than cucumber- or ammonia-baited traps. Shelly and Kurashima [26] demonstrated that the torula yeast borax trap captured a greater number of both sexes of *Z*. *cucurbitae* than the Cera Trap. Two GF-120 baits, one pulverized with 10% of ammonium carbonate and the second pulverized with ammonium acetate, were evaluated for their attraction of *R. pomonella*. The results demonstrated that bait pulverized with ammonium carbonate (10%) was found to be more attractive than bait pulverized with ammonium acetate, and the former suppressed a significantly greater population of *R. pomonella* [39].

Keeping the above facts in view, different PHS alone and in combination with other PHS lure sources (ammonium acetate, Di-ammonium phosphate, and acetic acid) were evaluated to find better attractants for both sexes of *B. zonata* under laboratory as well as field conditions on the basis on attractancy.

## 2. Materials and Methods

### 2.1. Rearing and Sampling Procedures of Bactrocera zonata

Fruits infected with *B. zonata* maggots were collected in plastic jars from Experimental Orchard, Square 9, University of Agriculture, Faisalabad (UAF), Pakistan (31.4303° N, 73.0672° E) and were brought to the Integrated Pest Management Laboratory. Plastic cages were filled with sterilized sand, and the infested fruits were kept in the cages. The ecological conditions within the cages were maintained at 26 ± 2 °C; 65 ± 5% R.H; and a 14 L:10 D photoperiod. The fully matured maggots leapt out of fruits while pupation occurred in the sand. A solution of 20% honey was provided to the adults of *B. zonata* as a food source. The egg laying was facilitated inside fresh and ripened fruits placed in hanging cups inside the rearing cages. The fresh fruits containing eggs were dissected and shifted to the new cages for the maintenance of the required progeny to be used for further experimentation.

### 2.2. Evaluation of Different PHS for Their Attractancy to Male and Female B. zonata

#### 2.2.1. Laboratory Study

Eleven PHS, including nine fruit extracts (pulp and juice of melon, banana, mulberry, mango, guava, apple, grapes, peach, and citrus), molasses, and protein hydrolysate (PH), were evaluated for their attractancy to *B. zonata* in comparison to GF-120 as a standard bait trap (SBT). The fruit extract of each fruit was prepared by blending it in an electric juicer–blender. The fruit extracts thus prepared were preserved in a deep freezer (−18 °C) for future use after adding preservatives (sodium benzoate and potassium sorbate). Molasses was obtained from Madina Sugar Mills Ltd., Faisalabad, Pakistan, while protein hydrolysate was purchased from STEDEC Technology, Pakistan.

**Figure 1:**
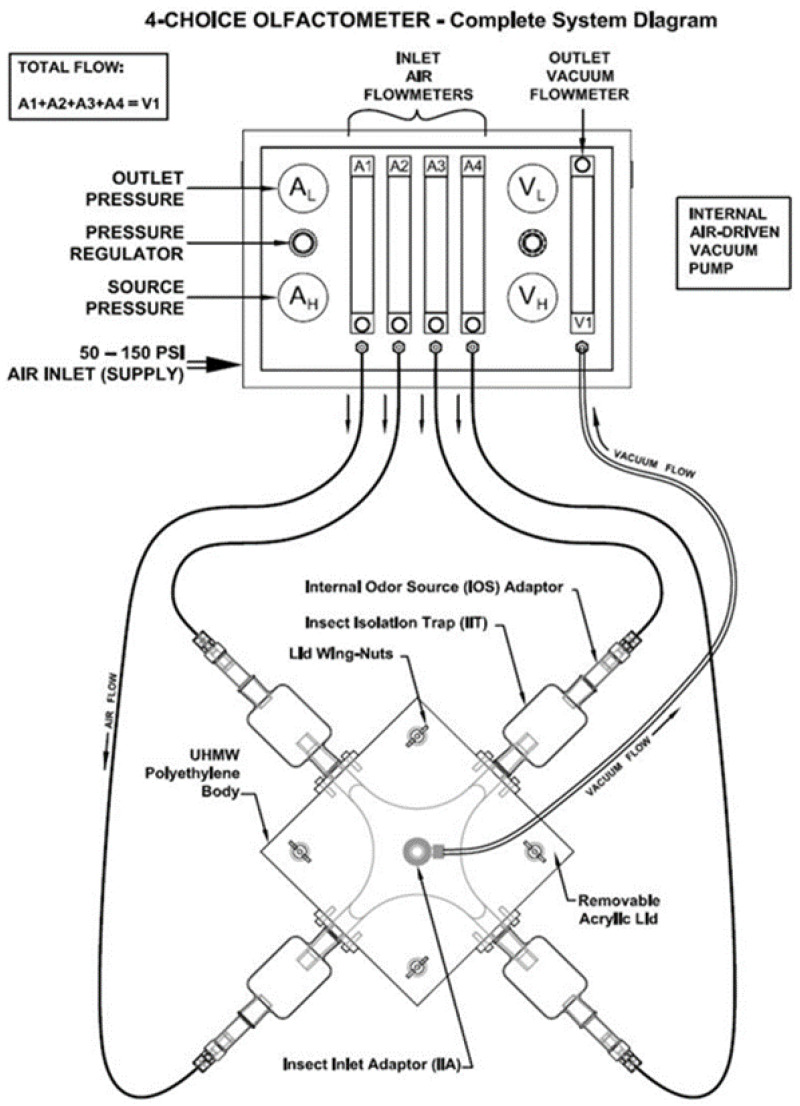
The primary choice arena of the olfactometer, measuring 30.48 × 30.48 × 2.54 cm, was topped with a removable lid. It included four outlet ports connected laterally to four odor source chambers and a ventral insect inlet port for introducing the test insect. Each lateral outlet port was linked to the internal odor source (IOS) using a glass insect isolation trap (IIT). The odor source was connected to an air delivery system that pumped moist air through the odor sources into the choice arena and used a vacuum to centralize airflow in the insect inlet chamber. The air delivery rate was 1 L/min. Air from all odor sources was directed to the insect inlet chamber using a vacuum suction mechanism, exposing the test insect to odors from different chambers, allowing it to make a choice. [Image source: Research Facility Solutions, LLC, Gainesville, Florida 32609 (https://rfsolutions.tech/index.html) (assessed on 4 June 2024) {formerly owned by ARS, Gainesville, Florida} (https://www.ars-fla.com/index.html) (assessed on 4 June 2024)].

Sponge cubes (7.5 × 7.5 × 7.5 cm) were used for the application of the PHS. Twelve sponge cubes were taken and soaked separately in PHS (each admixed with 1 mL of Spinosad) till the saturation of the sponge cubes. The PHS-saturated sponge cubes were separately placed in each small jars of the 4-choice olfactometer (TOP-4-150, Toption Group Co., Ltd., Yanta District, Xi’an, China) under laboratory conditions (26 ± 2 °C; 65 ± 5% R.H and 14 L:10 D photoperiod) (Figure 1).

Two hundred newly emerged adult male and female fruit flies were aspirated from each *B. zonata* culture and further grouped into four batches with fifty flies each. These batches were marked as Batch-A_1_ (50 *B. zonata* starved female), Batch-A_2_ (50 *B. zonata* starved male), Batch-B_1_ (50 *B. zonata* fed female), and Batch-B_2_ (50 *B. zonata* fed male). Batch-A_1_ and Batch-A_2_ were mixed in a small plastic jar and tagged as Starved-Batch (SB); while Batch-B_1_ and Batch-B_2_ were mixed in other separate small plastic jars and tagged as Fed-Batch (FB). The SB *B. zonata* were kept starved for 24 h, and the FB *B. zonata* were offered an adult fruit fly’s diet (honey, protein, and sugar mixture) for 24 h. The *B. zonata* of both batches were anesthetized by keeping both jars in the refrigerator (4 °C) till all flies became sluggish (10 min). The *B. zonata* of batch SB were marked green while the *B. zonata* of batch FB were marked red on the thorax with a permanent marker. After marking, the jars of both batches were kept at the ideal temperature (26 ± 1 °C) till all *B. zonata* became active, after which they were released into the olfactometer’s central large chamber. Before starting the new treatment, the olfactometer was washed with distilled water and allowed to air-dry completely at room temperature. Fresh *B. zonata* from both batches were used for the new treatment.

The attractiveness was evaluated with a completely randomized design (CRD) by counting the score of *B. zonata* attracted to each PHS. The experiment was repeated five times. The *B. zonata* attracted to the PHS were counted after 12 h, and the collected data were then transformed into an attractancy rating/index using the following formula [40].

$$Attractancy\;Index\;(AI)\;=\frac{Insects\;attracted\;by\;candidate\;PHS\;-\;Insects\;attracted\;by\;standard\;PHS}{Total\;insect\;attracted\;(\;candidate\;PHS\;+\;standard\;PHS)}\;\times \;100$$

On the basis of attractancy indices, the PHS were categorized into different classes [40] (Table 1).

#### 2.2.2. Field Study

Twelve PHS-saturated sponge cubes were prepared as described in the previous section and separately placed at the bottom of the traps (made up of plastic bottles, by cutting the top third of the bottle, inverting it to create a funnel, and securing it into the bottom part). The traps with PHS-saturated sponge cubes along with the SBT were installed in the center of the tree canopy of each plant, following the methodology of Nisar et al. [6], at an equal distance from the central stem of the tree. The study was laid out in mango orchards situated at the main campus of the University of Agriculture Faisalabad (UAF), Punjab, Pakistan (N 31°25′46.8048″, E 73°4′14.3112″), where the average daily temperature and relative humidity (average of day and night) fluctuated between 36 and 40 °C and 56 and 72% R.H, respectively, during the study period (June–July). The experiment was laid out in a randomized complete block design (RCBD), and the treatments (traps with PHS-saturated sponge cubes) were applied to five trees (one centermost tree and one of each eastward, westward, northward, and southward boundary trees). A 2 m trap-to-trap distance was maintained. The fruit flies attracted and trapped in each trap were collected on a daily basis for two weeks in plastic zip-bags and brought to the IPM laboratory. After collection of the trapped fruit flies, the traps were rotated one step, and this was repeated for two weeks. The collected fruit flies were separated into both sexes of *B. zonata* under a microscope in the IPM laboratory based on key characters [41] and were separately counted. The data collected on a daily basis for two weeks were transformed into *B. zonata* trapped/day/trap and then the attractancy rating/index was calculated [40].

### 2.3. Evaluation of Different Combinations of Highly Attractive PHS Admixed with Other PHS lure Sources in Various Ratios for Their Attractancy to Male and Female B. zonata

#### 2.3.1. Laboratory Study

This experiment consisted of baits of five phagostimulant-mixtures (PHS-Mix) with different ratios of PHS lures, such as casein, beef extract, cedar oil, yeast, fish extract, starch, mulberry, rose oil, protein hydrolysate, and molasses. These lure sources were mixed with banana squash in five different ratios, the details of which are given in Table 2. The fruit extract of each fruit was prepared by blending it in electric juicer–blender. The fruit extracts thus prepared were admixed with other ingredients and preserved in a deep freezer (−18 °C) for future use after adding preservative (potassium sorbate). Cubes of sponge (7.5 × 7.5 × 7.5 cm) were cut and used for the application of the PHS. Six sponge cubes were taken and soaked separately in PHS-Mix (each admixed with 1 mL of Spinosad) till the sponge cubes became saturated. These PHS-Mix-saturated sponge cubes were separately placed in each small jar of the olfactometer. Laterally, the same procedure was followed as that in the laboratory study with the olfactometer to evaluate the PHS for their attractancy to *B. zonata*.

#### 2.3.2. Field Study

Thirty PHS-Mix-saturated sponge cubes were prepared as described in the laboratory study and were separately placed at the bottom of the traps. The set of six traps with PHS-Mix-saturated sponge cubes were installed inside the canopy of the same tree at an equal distance from the central stem of the tree. The same procedure was followed as that in the field study to evaluate the PHS for their attractancy to *B. zonata*.

### 2.4. Evaluation of Different Combinations of Highly Attractive PHS-Mix Admixed with Ammonium Acetate, DAP, and Acetic Acid in Various Ratios for Their Attractancy to Male and Female B. zonata

#### 2.4.1. Laboratory Study

This experiment consisted of five phagostimulant-AdMixtures (PHS-AdMix) with different combinations of highly attractive PHS-Mix admixed with ammonium acetate, DAP, and acetic acid in various ratios. These components of the PHS-AdMix were admixed in five different ratios, the details of which are given in Table 3. The PHS-AdMix prepared were preserved in a deep freezer (−18 °C) for future use after adding preservative (potassium sorbate). Cubes of sponge (7.5 × 7.5 × 7.5 cm) were cut and used for the application of the PHS. Six sponge cubes were taken and soaked separately in PHS-AdMix (the PHS-AdMix each had 1 mL of Spinosad) till the sponge cubes became saturated. These PHS-AdMix-saturated sponge cubes were separately placed in each small jar of the olfactometer. The same procedure was followed as that in the laboratory study with the olfactometer to evaluate the PHS for their attractancy to *B. zonata*.

#### 2.4.2. Field Study

Thirty PHS-AdMix-saturated sponge cubes were prepared as described above and were separately placed at the bottom of the traps. The traps with PHS-AdMix-saturated sponge cubes were installed inside the canopy of the same tree at an equal distance from the central stem of the tree. The same procedure was followed as that in the field study to evaluate the PHS for their attractancy to *B. zonata*.

### 2.5. Statistical Analysis

The attractancy index data were analyzed for their main effects using one-way ANOVA through the multivariate general linear model (MGLM) technique using STATISTICA software version 10.0 to determine the parameters of significance and the mean values for the different treatments. The treatments with significant differences (recognized by *p* < 0.05) were subjected to Tukey’s honestly significant difference (HSD) test for paired comparisons at a probability level of 5% [42]. The means were also subjected to the Wilks’ lambda test to determine effective hypothesis decomposition based on the calculated *F*-value and *p*-value using STATISTICA software version 10.0. Similarly, using the same software, 95% confidence intervals (CIs) of the mean values were also computed to determine the upper and lower limits around the sample means and to visualize uncertainty in the summary points shown in the graphs. These CI values were then used to determine the variation in significance of the mean values of the treatments if their respective 95% CIs did not overlap.

## 3. Results

### 3.1. Evaluation of Different PHS for Their Attractancy to Male and Female B. zonata

#### 3.1.1. Laboratory Study

The ANOVA parameters revealed that the PHS had significant effects (*p* < 0.05) on the attractancy indices for the fed and starved batches of male and female *B. zonata* when the PHS were evaluated in the olfactometer under laboratory conditions (Table 4).

**Figure 2:**
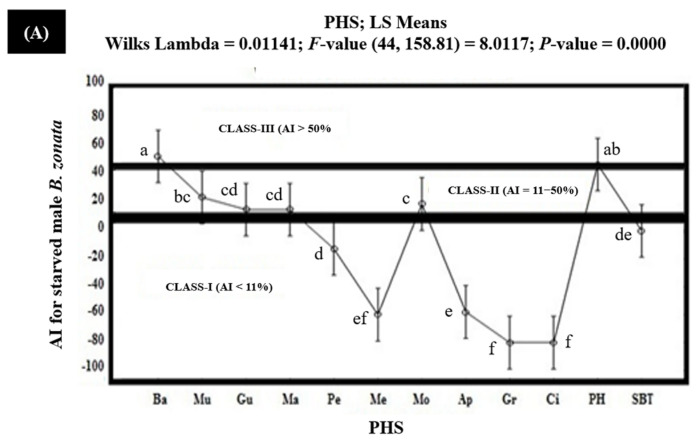

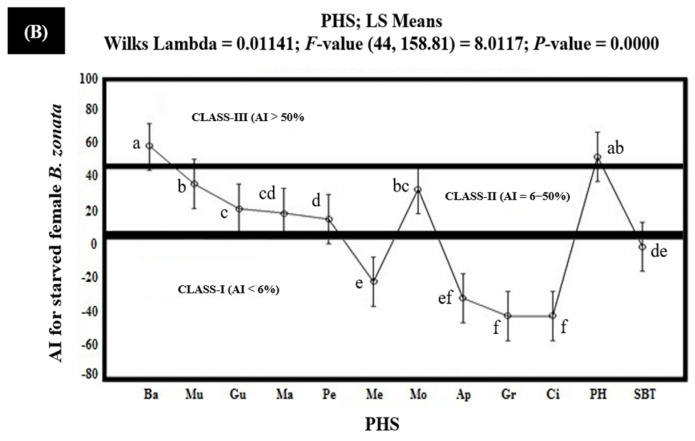
Attractancy indices (AIs) (%) of different PHS (Ba: Banana; Mu: Mulberry; Gu: Guava; Ma: Mango; Pe: Peach; Me: Melon; Mo: Molasses; Ap: Apple; Gr: Grapes; Ci: Citrus; PH: Protein Hydrolysate; SBT: Standard Bait Trap) for starved male (**A**) and female (**B**) *B. zonata* in olfactometer. AI of SBT (GF-120) is zero because it was used as a standard. Bars indicate 95% confidence intervals (as the ranges of upper and lower limits of CIs for mean values of each PHS did not overlap; so, AIs of PHS varied significantly from each other). Means sharing similar style letters do not significantly differ at probability level of 5%.

The results of the olfactometric studies in the laboratory for the starved batches of female and male *B. zonata* demonstrated that the banana extract exhibited 60.2% and 53.3% AIs for the starved female and male *B. zonata*, respectively, and proved to have strongly/highly attractive (Class-I) PHS (AI > 50%) for the starved female and male *B. zonata*. Protein hydrolysate exhibited 53.6% and 47.5% AIs for the starved female and male *B. zonata*, respectively, and proved to have strongly/highly attractive (Class-I) PHS (AI > 50%) for the starved female *B. zonata* but moderately attractive (Class-II) PHS (AI = 6–50%) for the starved male *B. zonata*. The extracts of mulberry (AI 37.3% for the female *B. zonata* and 23.9% for the male *B. zonata*), guava (AI 22.6% for the female *B. zonata* and 15.2% for the male *B. zonata*), mango (AI 20.0% for the female *B. zonata* and 15.2% for the male *B. zonata*), and molasses (AI 34.3% for the female *B. zonata* and 19.2% for the male *B. zonata*) exhibited AIs ranging between 6 and 50% and proved to have moderately attractive (Class-II) PHS for the female and male *B. zonata*. The extract of peach exhibiting a 16.4% AI proved to have moderately attractive (Class-II) PHS for the female *B. zonata* while the non-attractive (Class-I) PHS demonstrated a −12.9% AI for the male *B. zonata*. The rest of the PHS (melon, apple, grapes, and citrus) demonstrated AIs ranging from −12.9% to −80% and proved to have non-attractive (Class-I) PHS for the female *B. zonata*. The negative sign with these AIs indicated that the attractancy of these PHS was significantly lower than the SBT (GF-120) (Figure 2).

**Figure 3:**
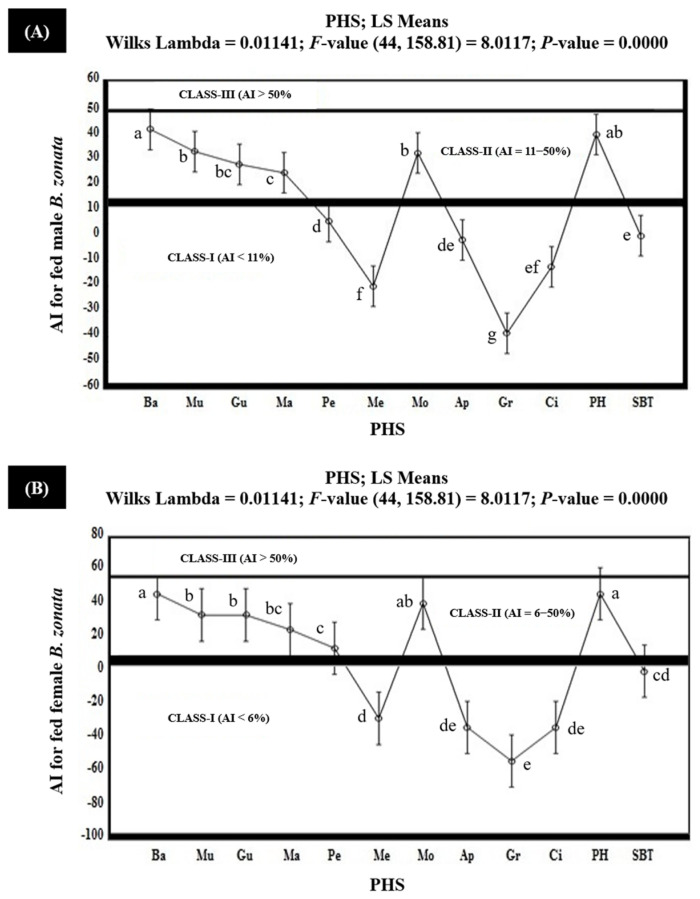
Attractancy indices (AIs) (%) of different PHS (Ba: Banana; Mu: Mulberry; Gu: Guava; Ma: Mango; Pe: Peach; Me: Melon; Mo: Molasses; Ap: Apple; Gr: Grapes; Ci: Citrus; PH: Protein Hydrolysate; SBT: Standard Bait Trap) for fed male (**A**) and female (**B**) *B. zonata* in olfactometer. AI of SBT (GF-120) is zero because it was used as a standard. Bars indicate 95% confidence intervals (as the ranges of upper and lower limits of CIs for mean values of each PHS did not overlap; so, AIs of PHS varied significantly from each other). Means sharing similar style letters do not significantly differ at probability level of 5%.

The results of the olfactometric study under laboratory conditions indicated that none of the PHS proved to be highly attractive (Class-III with AI > 50%) to the fed batches of both male and female *B. zonata*. The extracts of banana, mulberry, guava, mango, molasses, and protein hydrolysate exhibited 46.0% and 42.1%; 33.3% and 37%; 33.3% and 22.6%; 24.7% and 20.0%; 40.5% and 32.8%; and 46.0% and 40.1% attractancy indices (AIs) for the fed batches of female and male *B. zonata*, respectively. These results reveal that the extracts of these PHS proved to be moderately attractive (AI = 6–50% and Class-II) for the fed female and male *B. zonata*. The extract of peach exhibited 13.7% and 5.7% AIs for the fed batches of female and male *B. zonata*, respectively, and proved to be moderately attractive (AI = 6–50% and Class-II) for the female *B. zonata*, while it was non- or minimally attractive (AI < 6% and Class-I) for the male *B. zonata*. However, their attractancy was higher than the SBT (GF-120). The attractancy index of the aforementioned PHS was found to be comparatively higher for the female *B. zonata* than for the male *B. zonata.* However, the AIs of the rest of the PHS (extracts of melon, apple, grapes, and citrus) ranged from −53.3% to −1.7% for the fed batches of both male and female *B. zonata*. These ranges of AIs for the extracts of melon, apple, grapes, and citrus demonstrate that these PHS proved to be non- or minimally attractive (AI < 6% and Class-I); however, the negative values of the AIs here indicate that the attractiveness of these PHS was lower than the SBT (GF-120) (Figure 3).

#### 3.1.2. Field Study

The ANOVA parameters demonstrated that the PHS had significant effects (*p* < 0.05) on the attractancy index for female and male *B. zonata* when the PHS were evaluated under field conditions (Table 5).

**Figure 4:**
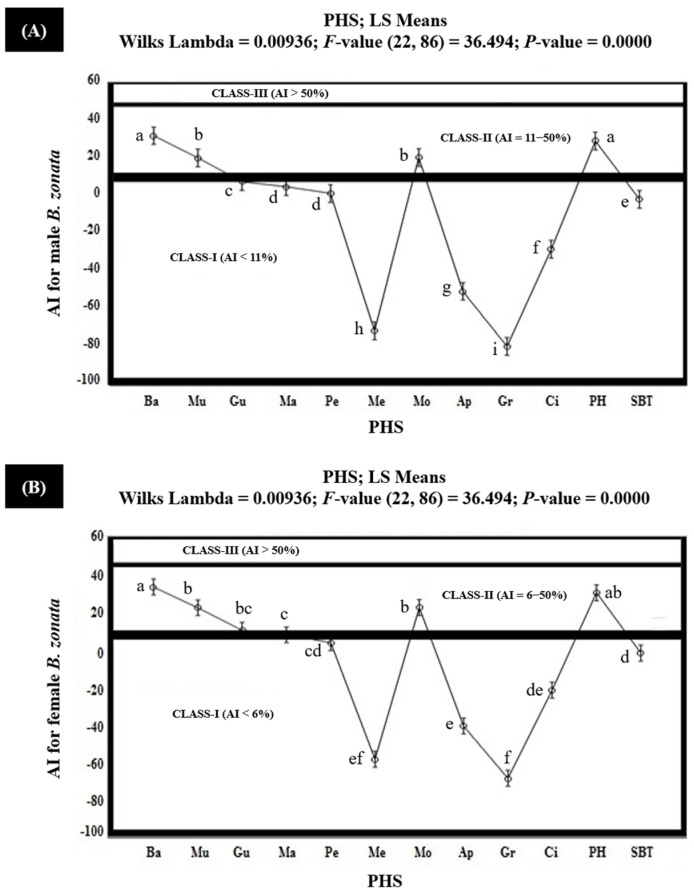
Attractancy indices (AIs) (%) of different PHS (Ba: Banana; Mu: Mulberry; Gu: Guava; Ma: Mango; Pe: Peach; Me: Melon; Mo: Molasses; Ap: Apple; Gr: Grapes; Ci: Citrus; PH: Protein Hydrolysate; SBT: Standard Bait Trap) for male (**A**) and female (**B**) *B. zonata* under field conditions. AI of SBT (GF-120) is zero because it was used as a standard. Bars indicate 95% confidence intervals (as the ranges of upper and lower limits of CIs for mean values of each PHS did not overlap; so, AIs of PHS varied significantly from each other). Means sharing similar style letters do not significantly differ at probability level of 5%.

The results of the field study showed that the banana extract demonstrated significantly higher AIs (35.5% for the female *B. zonata* and 32.7% for the male *B. zonata*) followed by protein hydrolysate (AIs of 32.6% for the female *B. zonata* and 29.8% for the male *B. zonata*), molasses (24.5% for the female *B. zonata* and 21.0% for the male *B. zonata*), mulberry (24.4% for the female *B. zonata* and 20.9% for the male *B. zonata*) and guava (12.2% for the female *B. zonata* and 8.2% for the male *B. zonata*). All these PHS exhibited AIs ranging between 6 and 50% in the attractancy scale and proved to have moderately attractive PHS (Class-II). However, the mango extract proved moderately attractive (Class-II) for female *B. zonata* (AI = 9.9%) but non-attractive (Class-I) for male *B. zonata* (AI = 5.6%). The peach extract exhibited 5.6% and 2.0% AIs for the female *B. zonata* and male *B. zonata*, respectively, and proved to have non-attractive (Class-I) PHS. The positive values of the AIs for peach indicated that its attractancy was higher than the SBT (GF-120). However, the rest of the PHS, including melon, apple, grapes, and citrus extracts, exhibited negative AIs ranging from −70.3% to −20.0% and also proved to have non-attractive (Class-I) PHS, but they were less attractive than the SBT (GF-120), as indicated by their negative AI values (Figure 4 and Table S1).

### 3.2. Evaluation of Different Combinations of Highly Attractive PHS Admixed with Other PHS lure Sources in Various Ratios for Their Attractancy to Male and Female B. zonata

#### 3.2.1. Laboratory Study

The ANOVA parameters explained that PHS-Mix had significant effects (*p* < 0.05) on the attractancy indices for the fed and starved batches of *B. zonata* when the PHS-Mix were evaluated in the olfactometer under laboratory conditions (Table 4).

**Figure 5:**
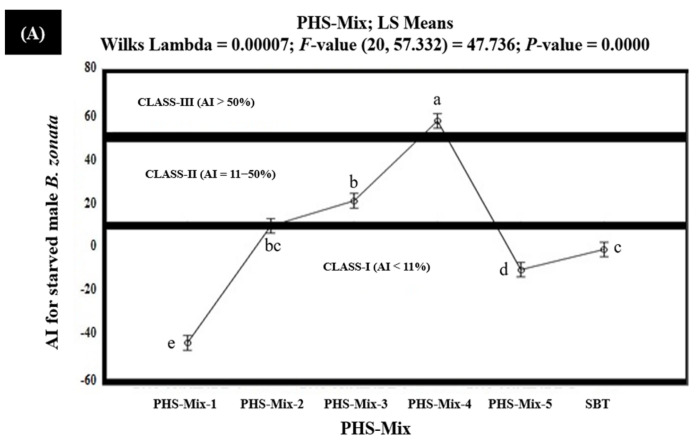

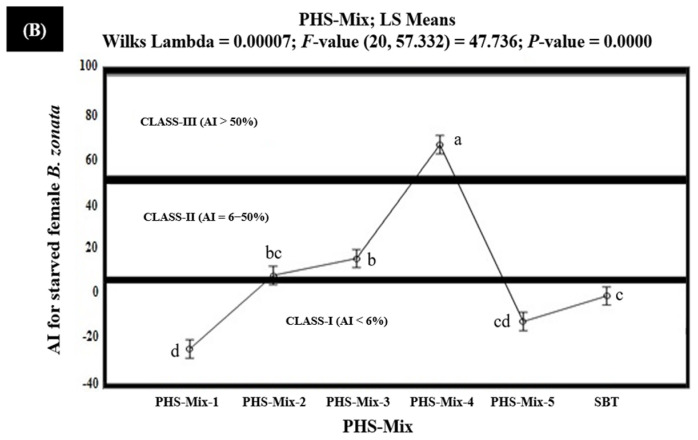
Attractancy indices (AIs) (%) of different PHS-Mix (PHS-Mix-1: Phagostimulants-Mixture-1; PHS-Mix-2: Phagostimulants-Mixture-2; PHS-Mix-3: Phagostimulants-Mixture-3; PHS-Mix-4: Phagostimulants-Mixture-4; PHS-Mix-5: Phagostimulants-Mixture-5; SBT: Standard Bait Trap) for starved male (**A**) and female (**B**) *B. zonata* in olfactometer. AI of SBT (GF-120) is zero because it was used as a standard. Bars indicate 95% confidence intervals (as the ranges of upper and lower limits of CIs for mean values of each PHS-Mix did not overlap; so, AIs of PHS-Mix varied significantly from each other). Means sharing similar style letters do not significantly differ at probability level of 5%.

**Figure 6:**
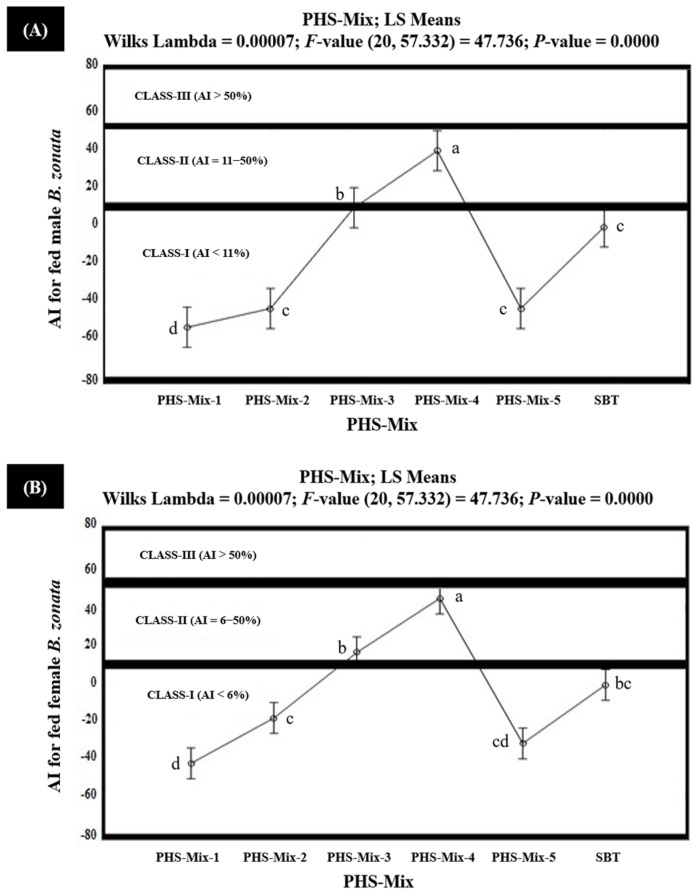
Attractancy Indices (AIs) (%) of different PHS-Mix (PHS-Mix-1: Phagostimulants-Mixture-1; PHS-Mix-2: Phagostimulants-Mixture-2; PHS-Mix-3: Phagostimulants-Mixture-3; PHS-Mix-4: Phagostimulants-Mixture-4; PHS-Mix-5: Phagostimulants-Mixture-5; SBT: Standard Bait Trap) for fed male (**A**) and female (**B**) *B. zonata* in olfactometer. AI of SBT (GF-120) is zero because it was used as a standard. Bars indicate 95% confidence intervals (as the ranges of upper and lower limits of CIs for mean values of each PHS-Mix did not overlap; so, AIs of PHS-Mix varied significantly from each other). Means sharing similar style letters do not significantly differ at probability level of 5%.

The results of the in vitro olfactometric study indicated that among the tested PHS-Mix, PHS-Mix-4 demonstrated strong attractiveness (Class-III with AI > 50%), exhibiting 68.6% and 59.5% AIs (AI > 50%) for the starved female and male *B. zonata,* respectively (Figure 5). However, PHS-Mix-4 exhibited 46.2% and 40.5% AIs for the fed male and female *B. zonata*, respectively, and proved to be moderately attractive (Class-II with AI 6–50%) for fed *B. zonata* (Figure 6). PHS-Mix-3 demonstrated AIs in the range of 10.2–22.5% and proved to be moderately attractive (Class-II with AI 6–50%) for both the starved and fed *B. zonata*. PHS-Mix-2 proved to be moderately attractive (Class-II with AI 6–50%), exhibiting 9.3% and 10.9 AIs for the starved female and male *B. zonata,* but the same demonstrated no or little attractiveness (Class-I with AI < 6%) for the fed female *B. zonata*, exhibiting −17.7% and −43.4% AIs, respectively. The negative AIs explained that PHS-Mix-2 exhibited 17.7% and 43.4% less attractiveness compared to the SBT (GF-120). PHS-Mix-1 and PHS-Mix-5 explained the AIs ranging from −24.0% to −53.3% and −9.3% to −43.4%, respectively, for both sexes and proved to be non- or minimally attractive (Class-I with AI < 6%) for the starved and fed *B. zonata*. These also demonstrate that PHS-Mix-1 and PHS-Mix-5 were found to be 24.0–53.3% and 9.3–43.4% less attractive to the starved and fed female *B. zonata* than the SBT (GF-120). Conclusively, PHS-Mix-4 was a significantly better PHS-Mix than PHS-Mix-3 (Figure 5 and Figure 6).

#### 3.2.2. Field Study

The ANOVA parameters demonstrated that the attractancy indices for female and male *B. zonata* varied significantly (*p* < 0.05) among PHS-Mix when these PHS-Mix were evaluated in field conditions (Table 5).

**Figure 7:**
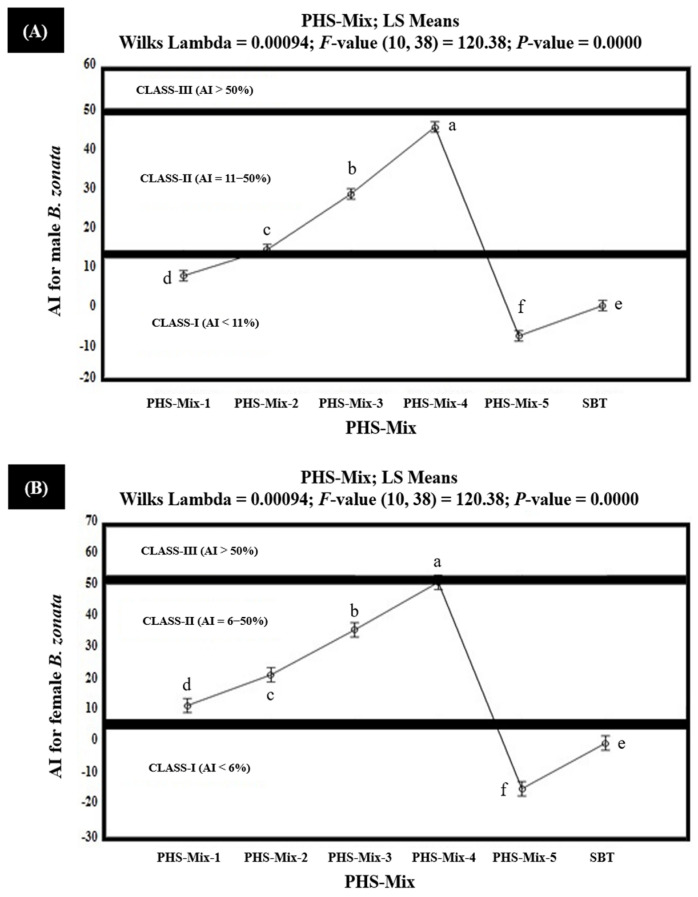
Attractancy indices (AIs) (%) of different PHS-Mix (PHS-Mix-1: Phagostimulants-Mixture-1; PHS-Mix-2: Phagostimulants-Mixture-2; PHS-Mix-3: Phagostimulants-Mixture-3; PHS-Mix-4: Phagostimulants-Mixture-4; PHS-Mix-5: Phagostimulants-Mixture-5; SBT: Standard Bait Trap) for male (**A**) and female (**B**) *B. zonata* under field conditions. AI of SBT (GF-120) is zero because it was used as a standard. Bars indicate 95% confidence intervals (as the ranges of upper and lower limits of CIs for mean values of each PHS-Mix did not overlap; so, AIs of PHS-Mix varied significantly from each other). Means sharing similar style letters do not significantly differ at probability level of 5%.

The results of the field study indicated that among the tested PHS-Mix, PHS-Mix-4 demonstrated strong attractiveness (Class-III with AI > 50%), exhibiting 51.2% AI for the female *B. zonata* but moderate attractiveness (Class-II with AI 6–50%), exhibiting 45.5% AI for the male *B. zonata*. PHS-Mix-3 demonstrated AIs in the range of 28.4–36.1% and proved to be moderately attractive (Class-II with AI 6–50%) for the female and male *B. zonata*. Similarly, PHS-Mix-2 proved to be moderately attractive (Class-II with AI 6–50%), exhibiting 21.8% and 14.2% AIs for the female and male *B. zonata*, respectively. PHS-Mix-1 also demonstrated 11.9% and 7.6% AIs for the female and male *B. zonata*, respectively, and exhibited moderate attractiveness (Class-II with AI 6–50%). Unexpectedly, PHS-Mix-5 explained the AIs ranging from −7.7% to −14.5% and proved to be non- or minimally attractive (Class-I with AI < 6%) for the female and male *B. zonata*. These also demonstrate that PHS-Mix-5 was found to be 7.7–14.5% less attractive to the female and male *B. zonata* than the SBT (GF-120). Conclusively, PHS-Mix-4 was significantly better than PHS-Mix, followed by PHS-Mix-3, PHS-Mix-2, and PHS-Mix-1 (Figure 7 and Table S2).

### 3.3. Evaluation of Different Combinations of Highly Attractive PHS-Mix Admixed with Ammonium Acetate, DAP and Acetic Acid in Various Ratios for Their Attractancy to Male and Female B. zonata

#### 3.3.1. Laboratory Study

The ANOVA parameters explained that PHS-AdMix had significant effects (*p* < 0.05) on the attractancy index for the fed and starved batches of male and female *B. zonata* when PHS-AdMix were evaluated in the olfactometer under laboratory conditions (Table 4).

**Figure 8:**
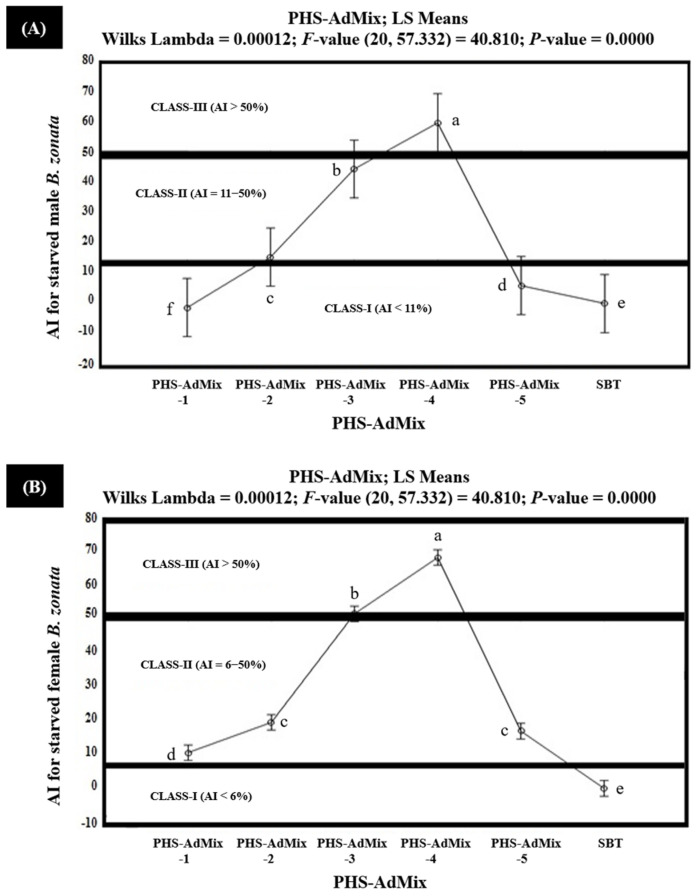
Attractancy indices (AIs) (%) of different PHS-AdMix (PHS-AdMix-1: Phagostimulants-AdMixture-1; PHS-AdMix-2: Phagostimulants-AdMixture-2; PHS-AdMix-3: Phagostimulants-AdMixture-3; PHS-AdMix-4: Phagostimulants-AdMixture-4; PHS-AdMix-5: Phagostimulants-AdMixture-5; SBT: Standard Bait Trap) for starved male (**A**) and female (**B**) *B. zonata* in olfactometer. AI of SBT (GF-120) is zero because it was used as a standard. Bars indicate 95% confidence intervals (as the ranges of upper and lower limits of CIs for mean values of each PHS-AdMix did not overlap; so, AIs of PHS-AdMix varied significantly from each other). Means sharing similar style letters do not significantly differ at probability level of 5%.

**Figure 9:**
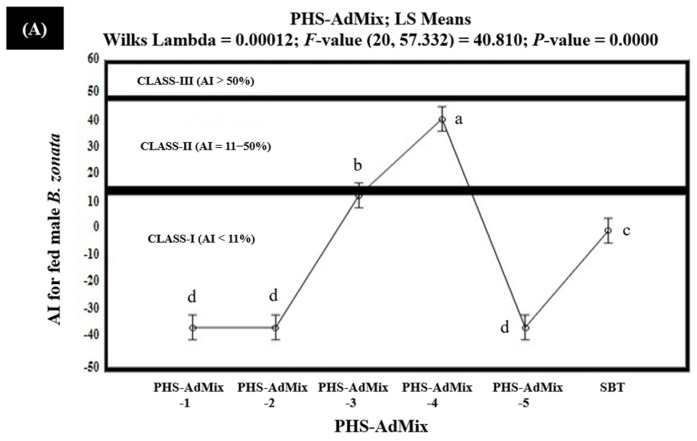

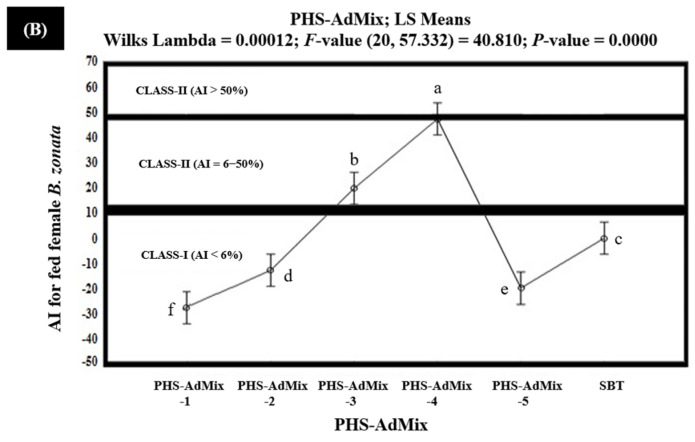
Attractancy indices (AI) (%) of different PHS-AdMix (PHS-AdMix-1: Phagostimulants-AdMixture-1; PHS-AdMix-2: Phagostimulants-AdMixture-2; PHS-AdMix-3: Phagostimulants-AdMixture-3; PHS-AdMix-4: Phagostimulants-AdMixture-4; PHS-AdMix-5: Phagostimulants-AdMixture-5; SBT: Standard Bait Trap) for fed male (**A**) and female (**B**) *B. zonata* in olfactometer. AI of SBT (GF-120) is zero because it was used as a standard. Bars indicate 95% confidence intervals (as the ranges of upper and lower limits of CIs for mean values of each PHS-AdMix did not overlap; so, AIs of PHS-AdMix varied significantly from each other). Means sharing similar style letters do not significantly differ at probability level of 5%.

The results of the in vitro olfactometric tests indicated that among the tested PHS-AdMix, PHS-AdMix-4 demonstrated strong attractiveness (Class-III with AI > 50%), exhibiting 70.7% and 64.4% AIs (AI > 50%) for the starved female and male *B. zonata,* respectively (Figure 8). However, PHS-AdMix-4 exhibited 41.3% and 36.6% AIs for the fed female and male *B. zonata,* respectively, and proved to be moderately attractive (Class-II with AI 6–50%) for the fed *B. zonata* (Figure 9).

PHS-AdMix-3 demonstrated AIs in the range of 13.1–44.9% and proved to be moderately attractive (Class-II with AI 6–50%) for the starved male and fed female *B. zonata*. However, PHS-AdMix-3 demonstrated 51.9% AI and proved to be strongly attractive (Class-III with AI > 50%) for the starved female *B. zonata*. PHS-AdMix-2, exhibiting 9.3% and 10.9 AIs, proved to be moderately attractive (Class-II with AI 6–50%) for the starved female and male *B. zonata*, but the same demonstrated no or little attractiveness (Class-I with AI < 6%) for the fed female and male *B. zonata*, exhibiting −12.7% and −32.9% AIs, respectively. The negative AIs explained that PHS-AdMix-2 exhibited 12.7% and 32.9% less attractiveness compared to the SBT (GF-120). PHS-AdMix-1 explained the 10.7% AI and proved to be moderately attractive (Class-II with AI 6–50%) for the starved female *B. zonata*. However, the same (PHS-AdMix-1) explained the AIs ranging from −35.9% to −1.4% and proved to be non- or minimally attractive (Class-I with AI < 11%) for the starved male *B. zonata* and the fed female and male *B. zonata*. These also demonstrate that PHS-AdMix-1 was found to be 1.4–35.9% less attractive to the starved male *B. zonata* and fed female and the male *B. zonata* than the SBT (GF-120). PHS-AdMix-5 explained the 17.0% AI and proved to be moderately attractive (Class-II with AI 6–50%) for the starved female *B. zonata*. However, the same (PHS-AdMix-5) demonstrated 5.3%, −19.8%, and −35.9% AIs for the starved male *B. zonata* and the fed female and male *B. zonata* and proved to be non- or minimally attractive (Class-I with AI < 6%) for these. The results also demonstrated that PHS-AdMix-5 was found to be 19.8 and 35.9% less attractive to the fed female and male *B. zonata* than the SBT (GF-120). Overall, PHS-AdMix-4 proved to be better than PHS-AdMix for the female and male *B. zonata* (Figure 8 and Figure 9).

#### 3.3.2. Field Study

The ANOVA parameters demonstrated that the attractancy indices for the female and male *B. zonata* varied significantly (*p* < 0.05) among PHS-AdMix when these PHS-AdMix were evaluated in field conditions (Table 5).

**Figure 10:**
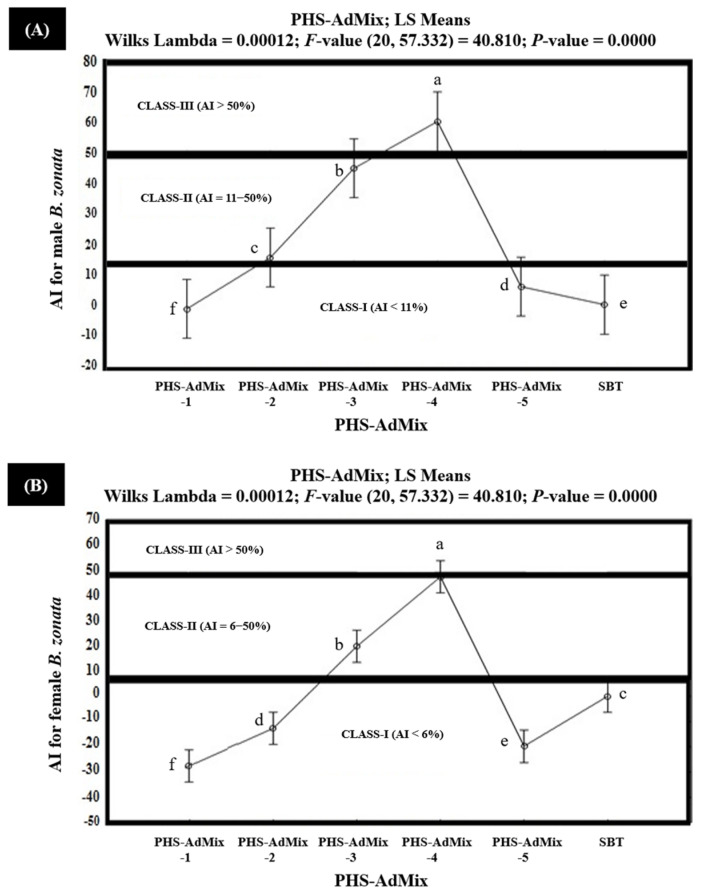
Attractancy indices (AIs) (%) of different PHS-AdMix (PHS-AdMix-1: Phagostimulants-AdMixture-1; PHS-AdMix-2: Phagostimulants-AdMixture-2; PHS-AdMix-3: Phagostimulants-AdMixture-3; PHS-AdMix-4: Phagostimulants-AdMixture-4; PHS-AdMix-5: Phagostimulants-AdMixture-5; SBT: Standard Bait Trap) for male (**A**) and female (**B**) *B.* zonata under field conditions. AI of SBT (GF-120) is zero because it was used as a standard. Bars indicate 95% confidence intervals (as the ranges of upper and lower limits of CIs for mean values of each PHS-AdMix did not overlap; so, AIs of PHS-AdMix varied significantly from each other). Means sharing similar style letters do not significantly differ at probability level of 5%.

The results of the field study indicated that among the tested PHS-AdMix, PHS-AdMix-4 demonstrated strong attractiveness (Class-III with AI > 50%), exhibiting 58.7% and 52.7% AIs for the female and male *B. zonata*. PHS-AdMix-3 demonstrated 50.7% AI and proved to be strongly attractive (Class-III with AI > 50%) for the female *B. zonata*. But the same (PHS-AdMix-3) explained 43.7% AI and proved to be moderately attractive (Class-II with AI 11–50%) for the male *B. zonata*. PHS-AdMix-2 proved to be moderately attractive (Class-II), exhibiting 32.9% and 23.6% AIs for the female and male *B. zonata*, respectively. Similarly, PHS-AdMix-1 proved to be moderately attractive (Class-II), exhibiting 25.9% and 18.3% AIs for the female and male *B. zonata*, respectively. Unexpectedly, PHS-AdMix-5 explained the 5.5% AI and proved to be non- or minimally attractive (Class-I with AI < 6%) for the female *B. zonata.* But the same (PHS-AdMix-5) demonstrated 6.6% AI and proved to be the least attractive (Class-I) for the male *B. zonata*. Overall, PHS-AdMix-4 proved to be better that PHS-AdMix, followed by PHS-AdMix-3, PHS-AdMix-2, and PHS-AdMix-1 under field conditions (Figure 10 and Table S3).

## 4. Discussion

### 4.1. Evaluation of Different PHS for Their Attractancy to Male and Female B. zonata

The attractiveness of the fruit flies depends on the level of satiation as well as the type and concentration of the fruit/food lure. The results of this experiment indicated that the fed *B. zonata* were less attracted to PHS compared to the starved *B. zonata*. This may be attributed to the fact that the fed batch had already been satisfied with food, which is why this batch demonstrated less attraction towards the PHS. The facts relating to little or no attraction of the fed fruit flies towards food lures has already been explained by a number of researchers [43,44,45,46]. The results of this experiment also demonstrated that banana was the most attractive PHS, followed by protein hydrolysate, molasses, mulberry, and guava. The difference in attractiveness of these PHS to both sexes of fruit fly species may be attributed to the variation in the concentration and types of primary and secondary volatile compounds emitted from the lure. Similar reasons have been explained by Prokopy and Reynolds [47], Bernays and Chapman [48], Brévault and Quilici [49], and Ren et al. [50] regarding variation in the attractiveness or deterrence of different food lures to fruit flies. The higher attraction of both sexes of fruit fly species to banana is attributed to the higher and strong concentration of highly attractive volatile compounds emitted from banana fruit. Ren et al. [50] also explained that the higher attraction of fruit flies to banana fruits was attributed to a particular aroma volatilized from the banana fruits. Similarly, Mowat et al. [51] also reported that the attraction of fruit flies to banana is due to a banana organic volatile (S)-2-Pentyl (R)-3-Hydroxyhexanoate. The results of this study about the higher attraction of banana are also in agreement with those of Sohail et al. [52], who concluded that banana proved to be a highly attractive and favorite food for *B. dorsalis* with the highest attraction and adult recovery (15%) due to its specific smell. The higher attraction of *B. zonata* to guava may be due to fact that it proves to be the most preferred fruit for oviposition, as explained by Sohail et al. [52], who documented the highest pupal recovery (103 pupae) from guava in a free-choice experiment. The attraction for oviposition of fruit flies, especially *Bactrocera* species, to guava fruit may be attributed to the fact that the offspring of fruit flies complete their growth and development more smoothly and easily in the shortest period on the pulp of guava [53,54]. Similarly, the moderate to strong attraction of fruit flies to protein hydrolysate and molasses in the current study may be attributed to the fact that just after eclosion and before oviposition, fruit flies visit protein and carbohydrate sources for egg maturation and energy metabolism. The higher attraction of fruit to GF-120 is due to the presence of protein hydrolysate and molasses, as reported by Vargas and Prokopy [55]. The results regarding the attractiveness of *B. zonata* to mulberry fruit cannot be compared and contrasted as information on the mulberry–tephritid association was not available in the literature. In conclusion, banana, PH, mulberry, and molasses can be utilized in the preparation of food baits for further experimentation.

### 4.2. Evaluation of Different Combinations of Highly Attractive PHS Admixed with Other PHS lure Sources in Various Ratios for Their Attractancy to Male and Female B. zonata

Protein sources saturation and the multicomponent components attraction system have been more attractive to fruit flies compared to the single-lure or double-lure attraction system [56]. In the second experiment there were five different ratios used to prepare the PHS-Mix, which consisted of banana squash, mulberry, protein hydrolysate, molasses, and various PHS lure sources, including beef extract, fish extract, yeast, starch, rose oil, casein, and cedar oil. Here are the PHS-Mix-1, PHS-Mix-2, PHS-Mix-3, PHS-Mix-4, and PHS-Mix-5 recipes: PHS-Mix-1: 1 part of all ingredients; PHS-Mix-2: 1 part banana and 0.75 parts of all the other ingredients; PHS-Mix-3: 1 part banana and 0.5 parts of the all other ingredients; PHS-Mix-4: 1 part banana and 0.25 parts of all the other ingredients; PHS-Mix-5: 1 part banana and 0.125 parts of all the other ingredients. According to the olfactometer and field studies, PHS-Mix-1 was found to be a Class-I unattractive PHS and a Class-II somewhat attractive one, respectively. Both the olfactometer and field tests showed that PHS-Mix-2 was moderately attractive to the starved batches. In both the laboratory and field experiments, PHS-Mix-5 was found to be unappealing to both the hungry and full populations of *B. zonata*. PHS-Mix-3 was classified as a Class-II (moderately attractive) PHS for both the starved and fed lots of female *B. zonata* in the olfactometer and field studies, while PHS-Mix-4 was determined to be the superior PHS-Mix. Overall, admixing of the different food lures improved attractiveness. The attractiveness of the PHS-AdMix was found stronger when the PHS were admixed in a moderate ratio. More attractiveness at a moderate ratio and less at a higher ratio may be attributed to the fact that volatiles of banana, which proved more attractive in the previous experiment, were in a higher concentration in PHS-Mix-4 and PHS-Mix-3 at the moderate ratio, and *B. zonata* were attracted more compared to the ratio of PHS-Mix-1 and PHS-Mix-2. Zeni et al. [57] also explained the similar reason behind the improved attractiveness of three component admixing system at a moderate ratio among the components. Nevertheless, it is not possible to compare and contrast the results of the current experiment due to lack of data in the literature reviewing the PHS-Mix used in the present study. However, some literature showed that the attractiveness of the bait was enhanced due to the addition of Casein in the bait [58]. In conclusion, PHS-Mix-4 was found to be the more attractive PHS-Mix and was used for further experimentation.

### 4.3. Evaluation of Different Combinations of Highly Attractive PHS-Mix Admixed with Ammonium Acetate, DAP and Acetic Acid in Various Ratios for Their Attractancy to Male and Female B. zonata

Ammonium compounds have been reported to enhance the attraction of fruit fly species of genus *Ceratitis* and *Bactrocera* [31,59,60]. In third experiment, five PHS-AdMix were prepared by mixing PHS-Mix-4 with ammonium acetate, Di-ammonium phosphate and acetic acid, i.e., PHS-AdMix-1 (1 part of all components), PHS-AdMix-2 (1 part of PHS-Mix-4 with 0.75 parts of all other components), PHS-AdMix-3 (1 part of PHS-Mix-4 with 0.5 parts of all other components), PHS-AdMix-4 (1 part of PHS-Mix-4 with 0.25 parts of all other components) and PHS-AdMix-5 (1 part of PHS-Mix-4 with 0.125 parts of all other components). PHS-AdMix-4 demonstrated strong attractiveness (Class-III) for staved female in olfactometer and field-female in field-studies of both *B. zonata;* however, the same exhibited moderate attractiveness (Class-II) for fed female *B. zonata.* Similarly, PHS-AdMix-3 proved strongly attractive (Class-III) only for starved-lot and field-lot of female *B. zonata* in olfactometer and field studies, respectively. While the rest of the PHS-AdMixtures demonstrated moderate (Class-II) to no attractiveness (Class-I) to both sexes of *B. zonata*. These results also indicated that the admixing of ammonium acetate, Di-ammonium phosphate and acetic acid with PHS-Mix enhanced their attractiveness for *B. zonata*. In the case of PHS-AdMix-4 and PHS-AdMix-3, the addition of ammonia sources (ammonium acetate, Di-ammonium phosphate and acetic acid) resulted in approximately two times higher attractiveness to female *B. zonata*. El-Gendy et al. [31], El-Metwally [59], Pimentel et al. [60] also reported that addition of ammonium compounds enhanced the attraction of fruit fly species of the genus *Ceratitis* and *Bactrocera*.

A large number of researchers evaluated the role of ammonium sources in enhancing the attractiveness of baits to fruit fly species and documented the positive association of ammonium and fruit fly attractiveness. For example, El-Gendy et al. [31] evaluated the attractiveness of ammonium acetate (AA), ammonium carbonate (AC), ammonium di-hydrogen orthophosphate (AD), di-ammonium hydrogen-orthophosphate (DA), and a mixture of AD and borax as a food lure for *B. zonata* adults under field conditions. Ammonium acetate demonstrated the highest attractancy to *B. zonata* adults, while admixing borax with AD improved the attractiveness of AD to *B. zonata* adults. Zeni et al. [57] documented the highest attraction of *Ceratitis capitata* and *Bactrocera zonata* to Buminal and ammonium compounds. Similarly, Pimentel et al. [60] reported ammonium acetate, putrescine, and trimethylamine as highly attractive biolures for *C. capitata.* According to Manrakhan et al. [56], an AA + putrescine + trimethylamine (TMA)-baited biolure attracted more female *C. capitata* (1494 female: 800 male) compared to AA+ TMA (764 female: 382 male) and AA+ putrescine (182 female: 82 male). Similar results were documented by Sookar et al. [34], who reported AA + putrescine + TMA as a highly attractive biolure for *B. zonata.* A biolure with a combination of acetic acid (AA), trimethylamine (TMA), and Buminal (B) exhibited 30.18–31.26% while AA + TMA demonstrated 21.70–26.04% trapping of *C. capitata* [61]. The results of the aforementioned researchers are highly in accordance with the results of the present study where the attractiveness of PHS-Mix-4 was improved by admixing it with ammonium acetate, di-ammonium phosphate, and acetic acid. However, the attractiveness of PHS-Mix-4 was stronger when it was admixed with a moderate concentration/ratio of ammonium acetate, di-ammonium phosphate, and acetic acid. This may be attributed to the fact that a very high or very low concentration of ammonia makes the lure less attractive to fruit flies [32]. These results can be compared with those of Thomas et al. [62], who reported that open-bottom plastic traps baited with a moderate ratio of ammonium acetate and putrescine (synthetic biolures) captured a higher population of *Anastrepha suspense* (Loew) and *A. ludens* (Loew) compared to a torula yeast-baited McPhail trap.

## 5. Conclusions

This study scientifically assessed the attractancy of several PHS and their combinations for both male and female *Bactrocera zonata* through laboratory and field bioassays. Remarkably, PHS-AdMix-4 emerged as a reliably strong attractant in both settings, representing high efficacy in attracting starved and fed *B. zonata*. The findings suggest that PHS-AdMix-4, when used in combination with the SBT (GF-120), holds important potential for practical application in integrated pest management approaches with good detection ability under field conditions; hence, PHS-AdMix-4 can also be utilized for phytosanitary surveillance programs in areas where this pest is not yet present. Its confirmed effectiveness in attracting *B. zonata* populations, particularly under field conditions, indicates its potential involvement in the development of targeted bait stations for controlling this agricultural pest and reducing its harmful effects on fruit crops. However, additional field trials and authentications are suggested to evaluate its performance across various environmental conditions and agricultural settings.

## Figures and Tables

**Table 1:** Attractancy indices of PHS for *B. zonata*.

Class	Attractancy Index		
	Male	Female	Response
I	<11	<6	Non or minimally attractive
II	11–50	6–50	Moderately attractive
III	>50	>50	Strongly attractive

**Table 2:** Treatments demonstrating different ratios of the ingredients in various PHS-Mix against *B. zonata*.

Treatment	Banana	Beef Extract	Fish Extract	Yeast	Starch	Rose Oil	Casein	Cedar Oil	Protein Hydrolysate	Mulberry	Molasses
PHS-Mix-1	1	1	1	1	1	1	1	1	1	1	1
PHS-Mix-2	1	0.75	0.75	0.75	0.75	0.75	0.75	0.75	0.75	0.75	0.75
PHS-Mix-3	1	0.5	0.5	0.5	0.5	0.5	0.5	0.5	0.5	0.5	0.5
PHS-Mix-4	1	0.25	0.25	0.25	0.25	0.25	0.25	0.25	0.25	0.25	0.25
PHS-Mix-5	1	0.125	0.125	0.125	0.125	0.125	0.125	0.125	0.125	0.125	0.125
SBT	-	-	-	-	-	-	-	-	-	-	-

**Table 3:** Treatments demonstrating different ratios among highly attractive PHS-AdMix, ammonium acetate, DAP, and acetic acid in various admixtures against *B. zonata*.

Treatments	Highly Attractive PHS-Mix from the Previous Experiment	Ammonium Acetate	Di-Ammonium Phosphate (DAP)	Acetic Acid
PHS-AdMix-1	1	1	1	1
PHS-AdMix-2	1	0.75	0.75	0.75
PHS-AdMix-3	1	0.5	0.5	0.5
PHS-AdMix-4	1	0.25	0.25	0.25
PHS-AdMix-5	1	0.125	0.125	0.125
SBT	-	-	-	-

**Table 4:** ANOVA parameters regarding attractancy indices of different PHS, PHS-Mix and PHS-AdMix to starved and fed batches of male and female *B. zonata* in olfactometer study (Replication = 5). ^a^ Degree of freedom of treatment; ^b^ Error degree of freedom; ^c^ Total degree of freedom; ** Highly significant at probability level of 5%.

SOV	*Df*	Starved Male *B. zonata*		Starved Female *B. zonata*		Fed Male *B. zonata*		Fed Female *B. zonata*	
		*F*-Value	*p*-Value	*F*-Value	*p*-Value	*F*-Value	*p*-value	*F*-Value	*p*-Value
PHS	11 ^a^/44 ^b^/59 ^c^	26.39558	0.000000 **	24.1528	0.000000 **	43.06331	0.000000 **	21.5759	0.000000 **
PHS-Mix	5 ^a^/20 ^b^/29 ^c^	445.5629	0.000000 **	263.617	0.000000 **	53.67452	0.000000 **	67.9933	0.000000 **
PHS-AdMix	5 ^a^/20 ^b^/29 ^c^	30.7374	0.000000 **	578.081	0.000000 **	218.1812	0.000000 **	82.6533	0.000000 **

**Table 5:** ANOVA parameters regarding attractancy indices of different PHS, PHS-Mix, and PHS-AdMix of male and female *B. zonata* under field conditions (Replication = 5). ^a^ Degree of freedom of treatment; ^b^ Error degree of freedom; ^c^ Total degree of freedom; ** Highly significant at probability level of 5%.

SOV	*df*	Female *B. zonata*		Male *B. zonata*	
		*F*-Value	*p*-Value	*F*-Value	*p*-Value
PHS	11 ^a^/44 ^b^/59 ^c^	275.3701	0.000000 **	268.317	0.000000 **
PHS-Mix	5 ^a^/20 ^b^/29 ^c^	952.654	0.000000 **	487.316	0.000000 **
PHS-AdMix	5 ^a^/20 ^b^/29 ^c^	1880.21	0.000000 **	145.755	0.000000 **

## Data Availability

The original contributions presented in the study are included in the article, further inquiries can be directed to the corresponding authors.

## Supplementary Materials

The following supporting information can be downloaded at: https://www.mdpi.com/article/10.3390/insects15070470/s1, Table S1: Number of male and female *B. zonata* collected from different PHS treatments under field conditions. Means sharing similar style letters do not significantly differ at probability level of 5%; Table S2: Number of male and female *B. zonata* collected from different PHS-Mix treatments under field conditions. Means sharing similar style letters do not significantly differ at probability level of 5%; Table S3: Number of male and female *B. zonata* collected from different PHS-AdMix treatments under field conditions. Means sharing similar style letters do not significantly differ at probability level of 5%.
