# Supplementary material for: Do Phagostimulants, Alone or Combined with Ammonium Acetate, Di-Ammonium Phosphate, and Acetic Acid, Effectively Attract Both Sexes of Peach Fruit Flies, Bactrocera zonata (Diptera: Tephritidae)?: Insights from Laboratory and Field Bioassays
Source: Insects. 2024 Jun 24;15(7):470. doi: 10.3390/insects15070470 (PMC11276886; doi:10.3390/insects15070470)
Supplement: Supplementary file 1 [file insects-15-00470-s001.zip › insects-3017038-supplementary.pdf]

The results of number of male and female *B. zonata* collected from different PHS treatments under field conditions demonstrated that maximum female and male *B. zonata* were collected from Banana (21.40 and 20.00 respectively) and PH (20.00 and 18.80 respectively), and results were significantly at par, while minimum female and male *B. zonata* were collected from Melon (2.80 and 1.80 respectively) and Graphs (2.00 and 1.20 respectively), and results were significantly at par (Table S1).

Table S1: Number of male and female *B. zonata* collected from different PHS treatments under field conditions. Means sharing similar style letters do not significantly differ at probability level of 5%

PHS	Female *B. zonata*	Male *B. zonata*
Banana	$21.40^{a} \pm 0.87$	$20.00^{a} \pm 0.75$
Mulberry	$16.80^{b} \pm 0.82$	$15.60^{b} \pm 0.74$
Guava	$13.00^{c} \pm 0.55$	$12.00^{c} \pm 0.55$
Mango	$12.40^{cd} \pm 0.54$	$11.40^{cd} \pm 0.54$
Peach	$11.40^{de} \pm 0.54$	$10.60^{cd} \pm 0.47$
Melon	$2.80^{h} \pm 0.22$	$1.80^{g} \pm 0.22$
Molasses	$16.80^{b} \pm 0.76$	$15.60^{b} \pm 0.67$
Apple	$4.40^{g} \pm 0.23$	$3.40^{f} \pm 0.23$
Grapes	$2.00^{h} \pm 0.18$	$1.20^{g} \pm 0.12$
Citrus	$6.80^{f} \pm 0.34$	$5.80^{e} \pm 0.34$
PH	$20.00^{a} \pm 0.86$	$18.80^{a} \pm 0.76$
SBT	$10.20^{e} \pm 0.50$	$10.00^{d} \pm 0.55$

The results of number of male and female *B. zonata* collected from different PHS-Mix treatments under field conditions demonstrated that maximum female and male *B. zonata* were collected from PHS-Mix-4 (22.40 and 19.20 respectively), while minimum female and male *B. zonata* were collected from PHS-Mix-5 (5.40 and 6.20 respectively) (Table S2).

Table S2: Number of male and female *B. zonata* collected from different PHS-Mix treatments under field conditions. Means sharing similar style letters do not significantly differ at probability level of 5%

PHS-Mix	Female *B. zonata*	Male *B. zonata*
PHS-Mix-1	$9.20^{d} \pm 0.50$	$8.40^{cd} \pm 0.43$
PHS-Mix-2	$11.20^{c} \pm 0.50$	$9.60^{c} \pm 0.47$
PHS-Mix-3	$15.40^{b} \pm 0.83$	$13.00^{b} \pm 0.75$
PHS-Mix-4	$22.40^{a} \pm 1.18$	$19.20^{a} \pm 0.96$
PHS-Mix-5	$5.40^{f} \pm 0.29$	$6.20^{e} \pm 0.34$
SBT	$7.20^{e} \pm 0.34$	$7.20^{de} \pm 0.34$

The results of number of male and female *B. zonata* collected from different PHS-AdMix treatments under field conditions demonstrated that maximum female and male *B. zonata* were collected from PHS-Mix-4 (27.60 and 23.20 respectively), while minimum female and male *B. zonata* were collected from PHS-Mix-5 (8.00 and 8.20 respectively) (Table S3).

Table S3: Number of male and female *B. zonata* collected from different PHS-AdMix treatments under field conditions. Means sharing similar style letters do not significantly differ at probability level of 5%

PHS-AdMix	Female *B. zonata*	Male *B. zonata*
PHS-AdMix-1	$12.20^{c} \pm 0.50$	$10.40^{c} \pm 0.43$
PHS-AdMix-2	$14.20^{c} \pm 0.50$	$11.60^{c} \pm 0.47$
PHS-AdMix-3	$22.00^{b} \pm 1.05$	$18.40^{b} \pm 0.89$
PHS-AdMix-4	$27.60^{a} \pm 1.09$	$23.20^{a} \pm 1.09$
PHS-AdMix-5	$8.00^{d} \pm 0.32$	$8.20^{d} \pm 0.34$
SBT	$7.20^{d} \pm 0.34$	$7.20^{d} \pm 0.34$
